# Bozeman on the Application of the Button Suture to Varicose Veins

**Published:** 1860-08

**Authors:** 


Bozeman on the Application of the Button Suture to Varicose Veins.

Pamphlet literature is acquiring an immense extension in the medical and surgical professions, and bids fair ere long to rival in importance the medical journals themselves.

We approve of the plan. The diffusion of knowledge will thereby be favored, and though many of the pamphlets will, like this, not be of great importance, yet many valuable ideas and suggestions will make their way into real usefulness, which otherwise had remained unnoticed in the brain of their inventors.

The pamphlet before us contains an article from the *N. O. Medical Journal*, by Dr. Bozeman, of Alabama, already known to the profession as having improved Sim's operations for vesico-vaginal fistula, by the invention of the button suture.

The application of the same suture is proposed for the obliteration of varicose veins.

The button suture of Dr. Bozeman, consists of fine silver wire, such as was prepared by Dr. Sims, which is inserted through the tissues wherever desired, and the two ends drawn through a small hole in a lead disc or button. The button thus closes up the suture by being pushed down to the skin, and is retained there by a perforated shot which is slipped on after it, and compressed upon the wire so as to hold it in position. The wire is then cut off near the shot and the suture is completed. In vesico-vaginal fistula, Dr. Bozeman makes several perforations in the same button to receive the several wires. In applying the suture to varicose veins of the *limbs*, Dr. B. inserts the wire upon one side of the vein, and brings it out at a separate opening upon the other, thus compressing a piece of skin with the vein.

It would probably be better after encircling the vein to return the wire through the *same opening* of the integument, and thus compress the vein alone, which would result in less irritation and pain.

In varicocele the author adopts this plan. He details three cases of varicose veins of the leg, and one case of varicocele successfully treated in this way.

The advantage of silver wire over silk ligature of the same strength, is its smaller size and its smoothness, by which it lies in its place with far less irritation than silk.

We think that there is a mistake in selecting silver in preference to gold or platina in such operations as vesico-vaginal fistula. Silver is readily reduced to a sulphuret by contact with the tissues, hence, in such situations it becomes blackened and looses its polish, and is more liable to irritate and cause suppuration than gold. We give the latter, therefore, the preference, both in fistula and in varicose veins. The use of the button, however, is a valuable improvement, and worthy of adoption in a variety of circumstances. Another point worthy of consideration in the button suture, is whether the button ought not to be of the same metal as the wire. The use of silver, gold, or platina, in contact with lead, establishes at once a minute galvanic battery, whose current acting on the tissue at the point of insertion, may in some cases serve to impede the process of adhesion.

